# Two Cases of Pediatric Common Peroneal Nerve Palsy Associated With Intermittent Pneumatic Compression

**DOI:** 10.7759/cureus.78650

**Published:** 2025-02-06

**Authors:** Hiroaki Urata, Kazunori Aoki, Hiroshi Sakihama, Hiroshi Kurosawa

**Affiliations:** 1 Department of Pediatric Critical Care Medicine, Hyogo Prefectural Kobe Children’s Hospital, Kobe, JPN

**Keywords:** body position, intermittent pneumatic compression, pediatric intensive care unit, peroneal nerve palsy, weight loss

## Abstract

Common peroneal nerve palsy occurs as a result of compression of the fibular head and has been reported in adults as a complication of intermittent pneumatic compression (IPC), but not in children. In this report, we present two cases of common peroneal nerve palsy. The first occurred in a 13-year-old girl with sepsis and the second in a 12-year-old boy who underwent vocal cord fixation. In both cases, we suspected the involvement of the IPC performed to prevent venous thromboembolism (VTE) during pediatric intensive care unit admission. It is crucial to ensure proper sizing and fitting of the IPC devices to avoid covering the fibular head and apply appropriate decompression. Moreover, recognizing risk factors, such as body position and thinness, is essential for understanding the onset of common peroneal nerve palsy.

## Introduction

In recent years, there has been an increasing trend in the occurrence of venous thromboembolism (VTE) in children, with a study using the pediatric intensive care unit (PICU) databases from 11 facilities in North America showing an incidence of 0.74% among 6653 PICU admissions [1]. With growth, hemostatic mechanisms mature, and the risk of thrombosis increases in those aged 10 years and older [2]. In addition to age, risk factors include the presence of endotracheal intubation for mechanical ventilation, immobility, and neoplastic diseases [3]. An assessment of these factors helps identify cases with high thrombotic risk, where VTE prophylaxis is implemented [2]. Intermittent pneumatic compression (IPC) is a method used to prevent VTE, although its implementation may lead to common peroneal nerve palsy [4]. This condition occurs when the common peroneal nerve, which runs superficially beneath the skin at the fibular head, is compressed, resulting in foot drop and numbness or sensory impairment from the lower leg to the dorsum of the foot.

Although reports of IPC-related common peroneal nerve palsy have been observed in adults [4-6], there are no such reports in children. Here, we report two cases of IPC-related common peroneal nerve palsy in children.

## Case presentation

Case 1

A 13-year-old girl was undergoing chemotherapy for mature B-cell lymphoma. She was 153 cm tall, weighed 34.7 kg, and had a body mass index (BMI) of 14.8. Drugs causing peripheral neuropathy were not administered. During chemotherapy, bone marrow suppression persisted. On the 12th day, she presented with fever and hypotension and was admitted to the PICU due to septic shock. Upon admission, respiratory failure necessitated respiratory support with a high flow nasal cannula (HFNC). However, on the third day of admission, respiratory deterioration led to tracheal intubation and mechanical ventilation, with sedation and muscle relaxation using midazolam, fentanyl, and rocuronium. Elastic stockings and IPC (Kendall SCDTM Sequential Compression Comfort Sleeve, size M, Cardinal Health™, Dublin, US) were simultaneously initiated for VTE prophylaxis. Position changes and pressure relief were performed every two to three hours during mechanical ventilation, and prone positioning was implemented for respiratory physiotherapy from the fifth to the eighth day of admission. Throughout the treatment course, there was a weight loss of approximately 5 kg and her weight after extubation was 29.9 kg, with a BMI of 12.7. Extubation was performed on the 13th day of admission. After extubation, the child complained of discomfort in the left leg. Examination revealed decreased temperature sensation in the left lower leg, difficulty in dorsiflexion of the ankle, and a manual muscle testing (MMT) score of one for left toe movement. Left common peroneal nerve paralysis was diagnosed via nerve conduction tests. Rehabilitation therapy is currently underway. Seven months after onset, there was an improvement in the central side of the temperature sensation impairment, but some peripheral side impairment remained. Dorsiflexion of the left ankle was possible, with MMT improving to 4/5.　　

Case 2

A 12-year-old boy had a history of traumatic bilateral vocal cord paralysis. He was 156 cm tall and weighed 47.7 kg, with a BMI of 19.6. Despite right vocal cord lateralization surgery, the symptoms of airway narrowing persisted. Subsequently, left vocal cord lateralization surgery was performed, and the patient was admitted to the PICU for postoperative management. Tracheal intubation and mechanical ventilation were continued upon admission, and sedation and muscle relaxation were managed with midazolam, fentanyl, and rocuronium until the second day of admission. The use of elastic stockings and IPC (Kendall SCDTM Sequential Compression Comfort Sleeve, size M, Cardinal Health™, Dublin, US), initiated in the operating room for VTE prophylaxis, was continued. The supine and extended neck positions were maintained during surgery, and position changes and pressure relief were performed every two to three hours after PICU admission. Extubation was performed on the fourth day of admission. After extubation, the child complained of pain in the left lower leg and difficulty in moving the ankle. Examination revealed resting pain in the left lower leg, difficulty in ankle dorsiflexion, and an MMT score of one for left toe movement. Left common peroneal nerve paralysis was diagnosed via nerve conduction tests. The treatment involved use of orthoses and continuing rehabilitation. Mecobalamin was administered for nerve damage, and carbamazepine was administered for severe pain. Six months post-injury, pain in the dorsal aspect of the foot during walking persisted, but walking itself was smooth, and dorsiflexion of the ankle had improved to MMT 4.

## Discussion

We encountered two cases of common peroneal nerve palsy after IPC was implemented for VTE prevention. Jaffray et al. listed age, endotracheal intubation, mechanical ventilation, immobility, and malignant diseases as risk factors for VTE [3]. In both cases, elastic stockings and IPC were used for VTE prevention owing to factors such as age, sedation, muscle relaxation management, and, in case 1, hematologic malignancy.

The elastic stockings were appropriately sized in both cases, and it was considered unlikely that they contributed to the common peroneal nerve palsy. The Kendall SCD Sequential Compression Comfort Sleeve, size M (peroneal abdominal circumference less than 53.3 cm) was used for IPC. Although the peroneal abdominal circumference was not measured in these cases, the average peroneal abdominal circumference in adults is said to be 37 cm for men and 34 cm for women [7]. Both patients were thought to have shorter circumferences, suggesting that the small size (35.5 cm or less) may have been appropriate. Furthermore, Yoon et al. reported a significantly higher incidence of common peroneal nerve palsy in the IPC group than in the non-IPC group in a comparative study involving 501 cases involving liver transplantation surgery [8]. This suggests that the IPC may be a risk factor for common peroneal nerve palsy. Therefore, although IPC was necessary for VTE prevention, the possibility of common peroneal nerve palsy development as a result of inappropriately sized devices was considered.

Thinness is also a risk factor for common peroneal nerve palsy. In neuropathic cachexia, chronic malnutrition leads to subcutaneous tissue loss, thereby increasing the likelihood of local compression of the fibular head [9]. In patients admitted to the PICU, the management of intubation and sedation for muscle relaxation is often required. Therefore, the progression of thinness during treatment may increase the risk of common peroneal nerve palsy. Although the patient in case 1 was thin, with a BMI of 14.8 upon admission, further weight loss during the course of treatment may have exacerbated the onset of common peroneal nerve palsy.

Both patients developed left-sided common peroneal nerve palsy. Yoon et al. reported a high incidence of left-sided peroneal nerve palsy among patients who underwent liver transplantation surgery [8] and the cause was thought to be the long duration in the left lateral decubitus position, indicating a correlation with body position.

In both the cases discussed here, patients were subjected to tracheal intubation and mechanical ventilation. However, in our hospital, the ventilator is positioned on the left side of patients' heads. Therefore, because of handling issues related to the respiratory circuit, it may have been easier to adjust the position to the left lateral decubitus position. As such, despite performing position changes every two to three hours, this may have contributed to the development of left-sided common peroneal nerve palsy.

## Conclusions

In addition to selecting the appropriate size, it is crucial to ensure proper fitting and perform frequent decompression when using IPC devices. Proper placement is essential to avoid complications. Particular attention should be given to preventing excessive compression over the fibular head, as this can lead to nerve-related complications. Clinicians should also be aware that pediatric patients with severe illnesses, such as those admitted to the PICU, are at increased risk of developing common peroneal nerve palsy. This risk is further exacerbated by emaciation, which reduces the protective soft tissue over the nerve, making it more susceptible to compression injuries.
